# Vasoinhibin, an N-terminal Prolactin Fragment, Directly Inhibits Cardiac Angiogenesis in Three-dimensional Heart Culture

**DOI:** 10.3389/fendo.2017.00004

**Published:** 2017-01-20

**Authors:** Ryojun Nakajima, Eri Nakamura, Toshio Harigaya

**Affiliations:** ^1^Laboratory of Functional Anatomy, Faculty of Agriculture, Department of Life Sciences, Meiji University, Kawasaki, Japan

**Keywords:** vasoinhibin, prolactin, peripartum cardiomyopathy, angiogenesis, heart

## Abstract

Vasoinhibins (Vi) are fragments of the growth hormone/prolactin (PRL) family and have antiangiogenic functions in many species. It is considered that Vi derived from PRL are involved in the pathogenesis of peripartum cardiomyopathy (PPCM). However, the pathogenic mechanism of PPCM, as well as heart angiogenesis, is not yet clear. Therefore, the aim of the present study is to clarify whether Vi act directly on angiogenesis inhibition in heart blood vessels. Endothelial cell viability was decreased by Vi treatment in a culture experiment. Furthermore, expression of proangiogenic genes, such as vascular endothelial growth factor, endothelial nitric oxide synthase, and VE-cadherin, were decreased. On the other hand, apoptotic factor gene, caspase 3, and inflammatory factor genes, tumor necrosis factor α and interleukin 6, were increased by Vi treatment. In three-dimensional left ventricular wall angiogenesis assay in mice, Vi treatment also inhibited cell migration, neovessel sprouting, and growth toward collagen gel. These data demonstrate that Vi treatment directly suppresses angiogenesis of the heart and support the hypothesis that Vi induce PPCM.

## Introduction

Prolactin (PRL) is a hypophyseal polypeptide hormone in humans that consists of 199 amino acids, with a molecular weight of approximately 23 kDa. PRL has many functions in various species and has been mainly shown to play roles in lactation, maintenance of pregnancy, maternal behavior expression, and angiogenesis promotion. However, an atypical form of PRL, which is cleaved into N-terminal 16 kDa fragment, has been shown to have antiangiogenic functions. Since growth hormone and placental lactogen also exert antiangiogenic effects when cleaved two-thirds from the N-terminal residue (1), these antiangiogenic fragments were named Vasoinhibins (Vi) (2). Vi derived from PRL also induce apoptosis by caspase 3 (3) and NF-kB (4), inhibit endothelial proliferation (5) and migration (6), and impair vasodilation (7).

A recent report showed that the Vi derived from PRL mediate peripartum cardiomyopathy (PPCM) (8), which is defined as heart failure presenting between the last month of pregnancy and 5 months after delivery in mothers with no history of heart failure. The cardinal symptom is dilation of the left ventricle, and it can induce maternal death in severe cases. In a PPCM mouse model generated with cardiomyocyte-specific STAT3 knockout, cardiomyopathy is induced by the increase of left ventricular Vi (8). Moreover, Vi cause not only endothelial cell but also myocardial damage through microRNA-146a released from endothelial cells, which reduces cardiomyocyte metabolic activity (9).

However, the direct effect of Vi on heart blood vessels is not clear. The purpose of this study was to investigate the direct effect of Vi on cardiac angiogenesis.

## Materials and Methods

### Hormones

Recombinant Vi was produced in *Escherichia coli* and purified *via* its His tag (Kitayama Labes, Nagano, Japan) as described previously (10). This recombinant Vi consisted of the 1 to 145 amino acid residues of mouse PRL.

### Cell Culture

Human umbilical vein endothelial cells (HUVECs) and a rat cardiomyoblast cell line (H9c2) (11) were obtained from DS Pharma Biomedical (Osaka, Japan) and American Type Culture Collection (Manassas, VA, USA), respectively. HUVECs were maintained in Medium for Normal Human Vascular Endothelial Cells (Kohjin Bio, Saitama, Japan) supplemented with 2% penicillin/streptomycin (Thermo Fisher Scientific, Waltham, MA, USA), and H9c2 cells were maintained in Dulbecco’s Modified Eagle Medium (DMEM, Thermo Fisher Scientific) supplemented with 10% fetal bovine serum (Thermo Fisher Scientific) and 2% penicillin/streptomycin (Thermo Fisher Scientific) at 37°C under controlled humidity and 5% CO_2_ atmosphere.

### Cell Viability Assay

Cell viability was evaluated by 3-(4,5-dimethylthiazol-2-yl)-5-(3-carboxymethoxyphenyl)-2-(4-sulfophenyl)-2H-tetrazolium, inner salt (MTS) assay (CellTiter 96^®^ AQueous One Solution Cell Proliferation Assay, Promega, Madison, WI, USA). HUVECs and H9c2 cells were seeded to the wells of 96-well plates at 6 × 10^4^ and 1 × 10^4^ cells/cm^2^, respectively. After cell adhesion to the wells, the culture media were exchanged for media supplemented with 0.5, 5, and 50 nM Vi or phosphate-buffered saline (PBS) endotoxin-free (AdipoGen, San Diego, CA, USA) as the vehicles. After incubation of the plate for 8 or 24 h, MTS was added to each well. Then, after incubation of the plate for an additional 2 h (total 10 h), 10% SDS was added to each well to stop the reaction. Absorbance at 490 nm was measured using a micro plate reader (PerkinElmer, Waltham, NJ, USA).

### Gene Expression Assay

Human umbilical vein endothelial cells were seeded on a 60-mm dish at 1.0 × 10^4^ cells/cm^2^. After cell adhesion, the culture media were changed to media containing 50 nM Vi or its vehicle. After incubation for 0, 8, or 24 h, the cells were collected. Total RNA was extracted from these cells using an RNeasy mini kit (Qiagen, Limburg, The Netherlands). The total RNA was reverse transcribed into cDNA using a QuantiTect Reverse Transcription kit (Qiagen) according to the manufacturer’s protocol. The cDNA was then used as the template for real-time PCR with SYBR select master mix (Thermo Fisher Science) and the 7500 real-time PCR system (Thermo Fisher Science). Specific primers for each gene are presented in Table 1. The expression level of internal 18 S ribosomal RNA was used for reference to quantify the relative target gene expression levels. The mRNA levels were expressed as fold change relative to those of the 0-h culture sample.

**Table 1:** **Specific primers for each gene**.

Genes	Forward	Reverse	Product size (bp)
*RNA18S5*	GTGGAGCGATTTGTCTGGTTA	CGGACATCTAAGGGCATCAC	166
*eNOS*	CAGTTGCTGCCAGGTCTGAT	GCTGCTTTGCAGGTTTTCCA	88
*VE-cadherin*	ATGAGATCGTGGTGGAAGCG	TGTGTACTTGGTCTGGGTGAAG	84
*VEGFA*	CTCCACCATGCCAAGTGGTC	GGGTCTCGATTGGATGGCAG	151
*TNF*	CCTGCTGCACTTTGGAGTGA	GAGGGTTTGCTACAACATGGG	125
*IL6*	GTTCCTGCAGAAAAAGGCAAAGA	CAGGAACTCCTTAAAGCTGCG	121
*CASP3*	TGCATACTCCACAGCACCTG	TTTCAGCATGGCACAAAGCG	131

*RNA18S5, RNA 18 S ribosomal 5; eNOS, endothelial nitric oxide synthase; VEGFA, vascular endothelial growth factor A; TNF, tumor necrosis factor α; IL6, interleukin 6; CASP3, caspase 3*.

### Three-dimensional Collagen Gel Culture

Four-week-old female C57/BL6 mice were purchased from CLEA Japan (Tokyo, Japan). The hearts were removed immediately after cervical dislocation. The method of *in vitro* assay for angiogenesis of the heart was based on previous reports (12, 13). Each heart was washed with PBS, and the left ventricular wall was fractionalized on the order of 1 mm^3^ in ice-cold DMEM. A fragment was immediately embedded in collagen gel solution that consisted of 5% FBS, 2% penicillin/streptomycin, 1 mg/ml collagen (Nitta Gelatin, Osaka, Japan), reconstitution buffer (Nitta Gelatin), and 30 ng/ml mouse vascular endothelial growth factor-164 (mVEGF_164_, Cell Signaling Technology, Danvers, MA, USA) in DMEM. After gelation, the gel was covered with cover medium consisting of 5% FBS, 2% penicillin/streptomycin, and 30 ng/ml mVEGF_164_ in DMEM. The collagen gel solution and cover medium included 100 nM Vi or its vehicle in advance. Incubation was performed for 1 week at 37°C in a humidified, 3% O_2_, 5% CO_2_ atmosphere. The cover medium was changed every 2 days.

### Angiogenesis Assay in the Heart

After incubation, the fragments and gels were fixed in 4% paraformaldehyde and used for whole-mount lectin staining. After permeabilization with Triton X-100, the fragments and gels were immersed with Block Ace (DS Pharma Biomedical, Osaka, Japan) and incubated with endothelium-specific lectin [fluorescein griffonia (Bandeiraea) simplicifolia lectin I, isolectin B4; Vector Laboratories, Burlingame, CA, USA]. Cell nuclei were stained with SlowFade Gold antifade reagent with DAPI (Invitrogen, Waltham, CA, USA). Three-dimensional images were obtained using a confocal laser scanning microscope (Olympus, Tokyo, Japan). To evaluate angiogenesis, the number of migrated cells and neovessels and the lengths of neovessels were measured using ImageJ software (National Institutes of Health, Bethesda, MD, USA). The number of migrated cells was calculated by counting cells in the gel. The number of neovessels was calculated by counting lectin-positive vessels that sprouted from the left ventricular fragment toward the gel, and the lengths of neovessels were averaged for all vessel lengths in the gel. The proportion of lectin-positive cells was calculated the positive cell number divided by total cell number.

### Statistical Analysis

Numerical data are expressed as means ± SEM. Significance was evaluated using Student’s *t*-test for comparisons between means and defined as a *P*-value <0.05.

### Ethics Statement

The experiment of animal use was carried out in accordance with the recommendations of AVMA Guidelines for the Euthanasia of Animals: 2013 Edition, American Veterinary Medical Association, and the Institutional Animal Care and Use Committee of Meiji University that approved the protocol of this study (IACUC-13-0013).

## Results

**Figure 1:**
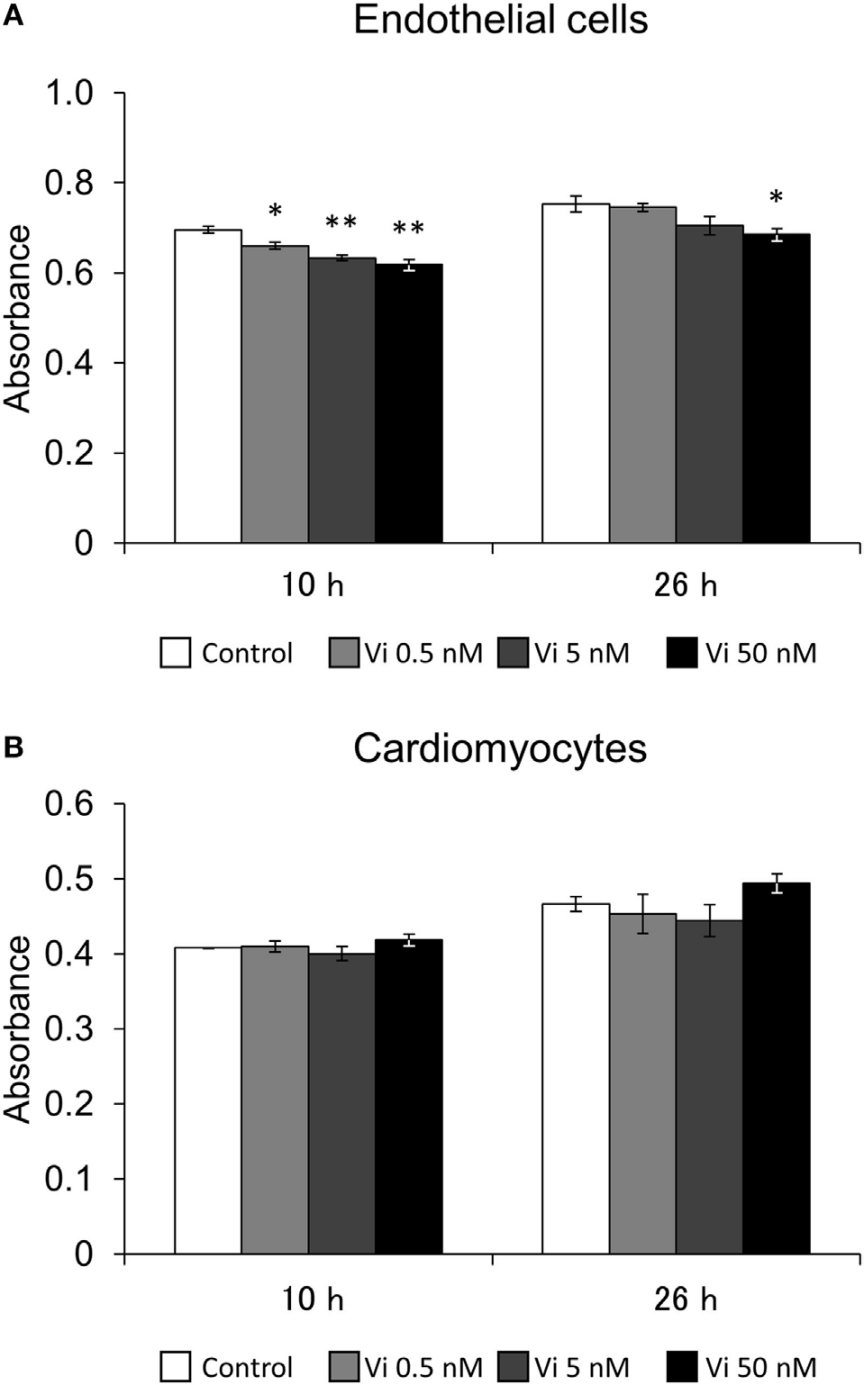
**Effects of vasoinhibins (Vi) on cell viability**. Effects of Vi on endothelial cell **(A)** and cardiomyocyte **(B)** viablity in 10- and 26-h culture. The white bar shows absorbance in the control. The light gray bar shows it in the 0.5 nM-Vi-treated group, the dark gray bar shows it in the 5 nM-Vi-treated group, and the black bar shows it in the 50 nM-Vi-treated group. Vi: vasoinhibin (*n* = 3, Mean ± SEM, *P < 0.05, **P < 0.01 vs. control).

In order to evaluate the effects of Vi on cell viability of endothelial cells and cardiomyocytes, an MTS assay was performed. After 10 h incubation, Vi from 0.5 to 50 nM significantly decreased endothelial cell viability compared with control. Furthermore, cell viability was significantly decreased in 50 nM Vi-treated endothelial cell after 26 h incubation (Figure 1A). On the other hand, Vi did not have any effect on cardiomyocyte viability (Figure 1B).

**Figure 2:**
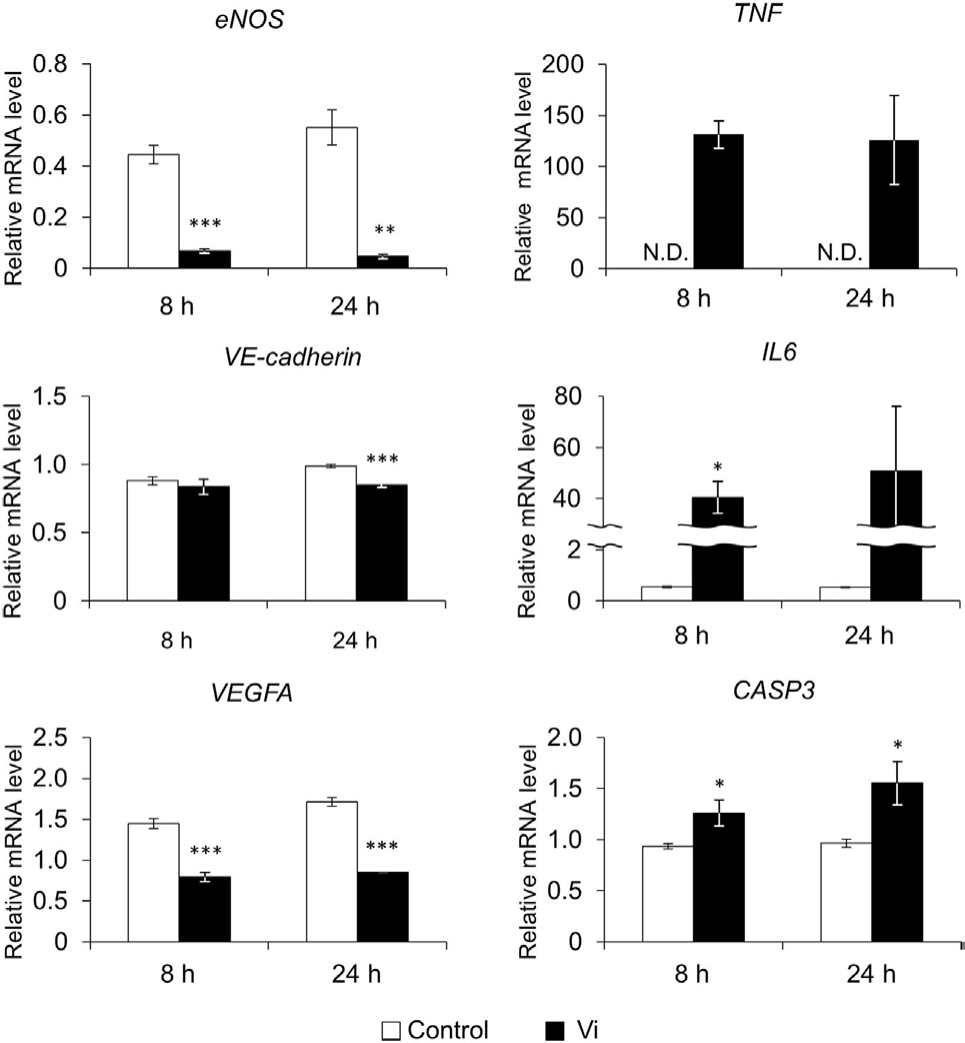
**Effects of vasoinhibins (Vi) on gene expression in endothelial cells by real-time PCR**. The white bar shows relative expression levels in the control and the black bar shows those in the Vi-treated group. eNOS, endothelial nitric oxide synthase; VEGFA, vascular endothelial growth factor A; TNF, tumor necrosis factor α; IL6, interleukin 6; CASP3, caspase 3; Vi, vasoinhibin 50 nM, N.D., not detectable (*n* = 3, Mean ± SEM, *P < 0.05, **P < 0.01, ***P < 0.001 vs. control).

To analyze the effect of Vi on mRNA expression of endothelial cells, real-time PCR was performed after 8- and 24-h incubation (Figure 2). Vi significantly decreased expressions of several genes related to angiogenesis (endothelial nitric oxide synthase; *eNOS, VE-cadherin*, and vascular endothelial growth factor A; *VEGFA*) compared with control. Caspase 3 (*CASP3*) gene expression, which indicates apoptosis, was significantly increased with Vi treatment compared with control. Although gene expression of tumor necrosis factor α (*TNF*) was not detected in control after 8- or 24-h incubation, neither Vi treatment induced the *TNF* expression. Interleukin 6 (*IL6*) expression was significantly higher with Vi treatment than with control on 8-h incubation.

**Figure 3:**
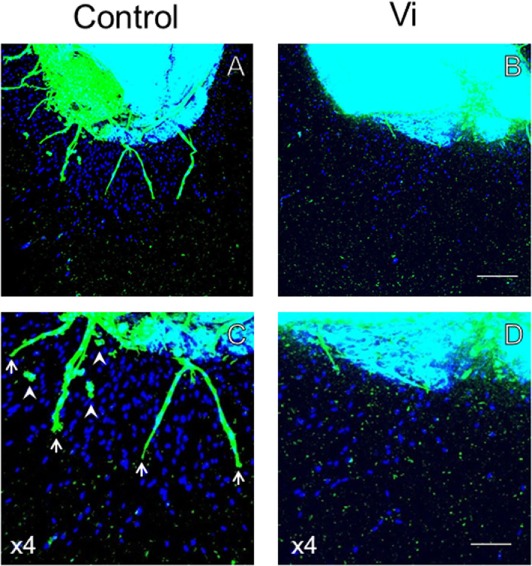
**Lectin staining of the left ventricular wall in three-dimensional culture**. Lectin staining of the three-dimensional cultured left ventricular wall using endothelial cell-specific lectin (green) and DAPI (blue). The heart segments were treated with VEGF under hypoxia in the absence or presence of Vi. Arrows indicate neovessel and arrow heads indicate migrated lectin-positive cell. **(A,B)** White bar = 200 μm. **(C,D)** White bar = 100 μm. Vi, vasoinhibin 100 nM.

**Figure 4:**
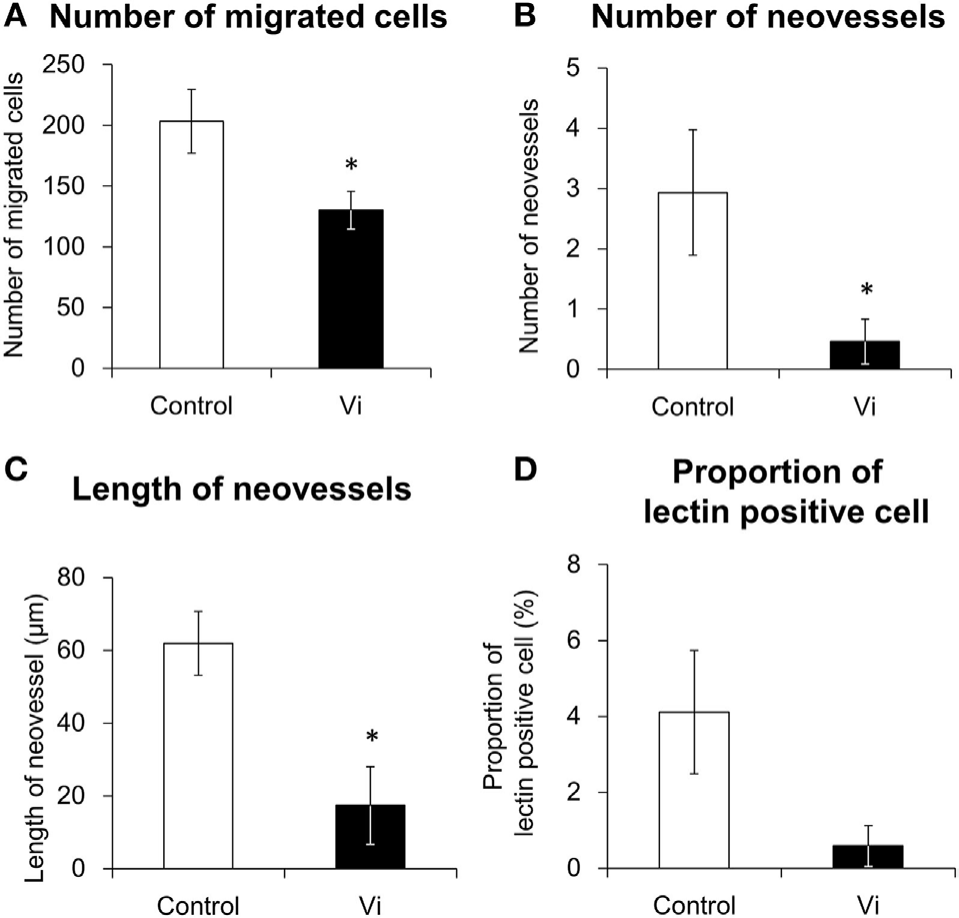
**Angiogenesis analysis of lectin-stained left ventricular wall in three-dimensional culture**. The white bar shows the numerical values in the control and the black bar shows those in the vasoinhibins (Vi)-treated group. The heart segments were treated with VEGF under hypoxia in the absence or presence of Vi. The procedures of measuring values are described in Section “Materials and Methods.” Vi, vasoinhibin 100 nM (*n* = 3, mean ± SEM, *P < 0.05 vs. control). **(A)** Number of migrated cells, **(B)** number of neovessels, **(C)** Length of neovessels, and **(D)** proportion of lectin-positive cell.

In order to analyze the antiangiogenic effects of Vi, three-dimensional heart fragment culture and angiogenesis assays were performed. The endothelial marker lectin-positive cells were seen in gel matrix and heart fragments (Figure 3). Migrations of endothelial marker-positive and -negative cells were observed. The neovessels consisted of endothelial marker-positive cells that were shown in gel matrix. To assess angiogenesis, the number of migrated cells and neovessels and the lengths of neovessels were measured. The number of migrated cells was significantly smaller with Vi than with control (Figure 4A). The number of neovessels was significantly less with Vi treatment than with control (Figure 4B). The length of neovessels was significantly shorter with Vi treatment than with control (Figure 4C). The proportion of lectin-positive cells was higher with Vi treatment than with control, but not significant (*P* = 0.07, Figure 4D).

## Discussion

In the present study, the anti-angiogenetic effect of Vi in the heart was observed. Vi decreased endothelial cell viability in the culture experiment, and it decreased expressions of proangiogenic genes, such as *VEGFA, eNOS*, and *VE-cadherin*. An apoptosis gene, *CASP3*, and inflammatory genes, *TNF* and *IL6*, were increased by Vi. Furthermore, Vi suppressed cell migration, vascular sprouting, and growth in the three-dimensional left ventricular wall angiogenesis assay.

Endothelial cell viability was decreased with Vi. On the other hand, Vi did not have any effect on cardiomyocyte viability. The endothelial cell is considered the main target cell of Vi because of its anti-angiogenetic effect. However, we previously reported that Vi had a proapoptotic effect on mammary epithelial cells (10). Therefore, Vi would exert effects not only on endothelial cells but also other cells. Since cardiomyocyte viability is decreased with adenovirus-induced Vi expression (8), the direct effect of Vi on cardiomyocyte viability was examined in this study. However, there was no obvious effect on its viability in the same experimental condition of endothelial cells. At least in part, the Vi concentration in media that reduced endothelial cell viability might not have been sufficient to decrease cardiomyocyte viability. In addition, since the MTS assay may measure not only cell viability but also metabolic activity and level of proliferation, further experiments would be required.

Vasoinhibins also altered expressions of genes that participated in anti-angiogenesis in the present study. Expression levels of *VEGFA, eNOS*, and *VE-cadherin* were decreased, and levels of *CASP3* was increased with Vi treatment. Especially, this is the first time that Vi are shown to downregulate both *VEGF* and *eNOS* expression in endothelial cells. VEGF is a potent angiogenic factor, and its angiogenic function is exerted by binding to VEGF receptor. Vi inhibits endothelial cell proliferation that is promoted by VEGFA (14). Since VEGF autocrine is necessary to vascular homeostasis (15), downregulation of VEGF expression by Vi may cause microvascular dysfunction. eNOS is one of the NOS isoforms that are constitutively expressed in endothelial cells. Nitric oxide, which is produced by eNOS, protects endothelial cells against apoptosis and promotes angiogenesis (16), and Vi inhibits eNOS activity through dephosphorylation (17). Moreover, Vi downregulates expression of inducible NOS, which is another NOS isoform (18). Likewise, we found that Vi downregulates *eNOS* expression in endothelial cell culture. VE-cadherin mediates cell adhesion, and is necessary for endothelial survival (19), and Vi downregulates the expression of *VE-cadherin* in the present study. In addition, the expression of *CASP3*, which is a key mediator of apoptosis, was increased with Vi treatment, in agreement with the previous study (20). These results confirm that the Vi also play a role in vascular anti-angiogenesis by the apoptotic involvement.

We consider that VEGF-receptor 2 (VEGF-R2) promote angiogenesis and Vi prevents its effects. VEGF increasesd eNOS expression by binding VEGF-R2 (21). The activation of eNOS was induced by increase of intracellular Ca^2+^ mobilization (22) and phosphorylation (23) through VEGF and VEGF-R2. In contrast, Vi inhibits both intracellular Ca^2+^ mobilization (7) and phosphorylation of eNOS (17). Moreover, Vi inhibits angiogenesis signaling through inhibiting Ras, which is one of the starting point of VEGF signaling (14).

It has been suspected that Vi is also involved to myocarditis, which was often observed in PPCM (24). In this study, inflammatory cytokines, *TNF* and *IL6*, were upregulated by Vi. Vi has a pro-inflammatory function *via* upregulation of intercellular adhesion molecule-1, vascular adhesion molecule-1, and E-selectin in endothelial cells (20). Moreover, expression level of eNOS is decreased by Vi in present study, and previous study shows that Vi inhibits eNOS activity. Since suppression of eNOS activity enhances microvascular permeability (25), there is a possibility that Vi directly induces myocarditis. In addition, TNF induces cardiomyocyte apoptosis (26), and IL6 induces hypertrophy and myocardial fibrosis (27). The blood levels of these cytokines were higher in PPCM patients (28). Therefore, Vi may cause cardiomyocyte damage through release of these cytokines, as well as microRNA-146a, which is released from endothelial cells by Vi and inhibits cardiomyocyte metabolic activity (9).

A previous study reported that left ventricular capillary density is decreased in the PPCM mouse model (8). It is known that healthy pregnant women develop heart hypertrophy with angiogenesis (29). The structural heart change during pregnancy is considered to be induced by not only VEGF but also by estrogen and cardiac volume overload (29). In this study, left ventricular angiogenesis was induced by the hypoxic condition and VEGF treatment. This induction of angiogenesis was inhibited by Vi treatment in three-dimensional heart culture.

In addition to endothelial cells, migration of non-endothelial lectin-negative cell was also observed in heart culture experiments. Thus, Vi may also inhibit migration of cells other than endothelial cells, such as pericytes and fibroblasts. Moreover, mature vessels that consist of multiple cells were not observed with Vi treatment in culture. Since Vi inhibits pericyte migration toward endothelial cells and vessel maturation (30), Vi also seems to play a inhibiting role in heart vessel maturation.

## Author Contributions

This study was designed by RN and carried out by RN and EN. RN and TH wrote the manuscript. All the authors have contributed to data collection or interpretation and critically reviewed the manuscript. The final manuscript was approved by all authors.

## Conflict of Interest Statement

The authors declare that the research was conducted in the absence of any commercial or financial relationships that could be construed as a potential conflict of interest. The reviewer BMS and handling Editor declared their shared affiliation, and the handling Editor states that the process nevertheless met the standards of a fair and objective review.
